# Biocompatibility and Carcinogenicity of Carbon Nanotubes as Biomaterials

**DOI:** 10.3390/nano10020264

**Published:** 2020-02-04

**Authors:** Kaoru Aoki, Naoto Saito

**Affiliations:** 1 Physical Therapy Division, School of Health Sciences, Shinshu University, 3-1-1 Asahi, Matsumoto, Nagano 390-8621, Japan; kin29men@shinshu-u.ac.jp; 2 Institute for Biomedical Sciences, Interdisciplinary Cluster for Cutting Edge Research, Shinshu University, 3-1-1 Asahi, Matsumoto, Nagano 390-8621, Japan

**Keywords:** carbon nanotubes, biocompatibility, carcinogenicity

## Abstract

With the development of nanotechnology in recent years, there have been concerns about the health effects of nanoparticles. Carbon nanotubes (CNTs) are fibrous nanoparticles with a micro-sized length and nano-sized diameter, which exhibit excellent physical properties and are widely studied for their potential application in medicine. However, asbestos has been historically shown to cause pleural malignant mesothelioma and lung cancer by inhalation exposure. Because carbon nanotubes are also fibrous nanotubes, some have raised concerns about its possible carcinogenicity. We have reported that there is no clear evidence of carcinogenicity by local and intravenous administration of multi-walled CNTs to cancer mice models. We firmly believe that CNTs can be a safe, new, and high-performance biomaterials by controlling its type, site of administration, and dosage.

## 1. Introduction

In recent years, nano-sized materials, with particle sizes ranging from 1 to 100 nm, have been used in various fields, and nanotechnology continues to make remarkable progress. These nanomaterials include carbon nanotubes (CNTs) and carbonaceous materials, such as fullerenes; metal particles, such as gold or silver nanoparticles and titanium oxide; ceramic particles, such as silicon dioxide and; organic polymer particles. The diverse applications of nanomaterials, include zinc and titanium oxides used in cosmetics [1,2]; nano Si cluster-SiO*_x_*-C composite material used in rechargeable lithium batteries [3], and; CNTs used in aircrafts and space vehicles. 

**Figure 1:**
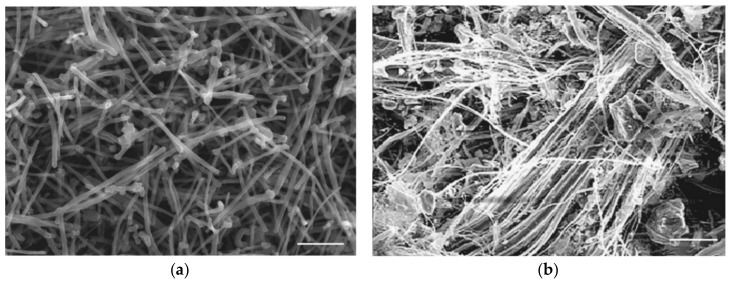
(**a**) Scanning electron microscopy (SEM) image of MWCNTs. Scale bar, 2 µm. (**b**) SEM image of asbestos (chrysotile). Scale bar, 20 µm. Reproduced with permission from [10]. Elsevier, 2010.

Carbon is an indispensable element for organic compounds, which is abundant in the living body and regarded for its excellent biocompatibility. In fact, carbon has already been used to improve the function of biomaterials, such as prosthetic heart valves and coronary stents [4,5]. CNTs are made of graphene sheets consisting of a series of carbon rings. These sheets are rolled into nano-sized cylindrical fibers (Figure 1a) that are light-weight with higher tensile strength than diamond. CNTs are widely researched for its potential industrial applications, due to their excellent electrical and thermal conductivity. The material is used in lithium-ion batteries to extend battery life and contribute to energy conservation. Furthermore, combining various materials with CNTs can reduce weight, while preserving strength; therefore, the material is being developed for applications in automobiles, aircrafts, ships, and space-crafts [6,7,8,9]. 

On the other hand, asbestos is a natural nanomaterial that was widely used in buildings from the 1950s to the 1970s for its exceptional heat insulation [10] (Figure 1b), fire resistance, and sound insulation; however, inhalation exposure to asbestos nanoparticles has been shown to cause malignant tumors, such as pleural mesothelioma, and their use was prohibited in various countries [11,12,13,14]. Inhaled asbestos nanoparticles are believed to cause long-term inflammation in the lungs, malignant transformation of mesothelial cells as a result of their reactivity, and formation of malignant mesothelioma [15]. 

CNTs have excellent material properties and can reinforce the mechanical strength of composite material, are used as a drug delivery system, and can potentially function as a high-performance biomaterial. However, CNTs are fibrous nanoparticles and may give many researchers the impression that the material is carcinogenic like asbestos. CNTs and asbestos often differ in their surface charge, hydrophilicity/hydrophobicity, active metal properties, tensile strength, and bio-durability. Despite these differences, the fibrous morphology of the nanoparticles has led some researchers to believe that CNTs may potentially be carcinogenic like asbestos [16]. The pharmacokinetics of these nanoparticles, as they are implanted in the body as biomaterials, remains unknown, and it is unclear how CNTs affect the living body. In this review, we will summarize currently reported results from biocompatibility and carcinogenicity tests on CNTs, in vitro and in vivo, and we will also discuss their effects on the living body, methods of evaluation, and potential as biomaterials.

## 2. Biocompatibility Testing of CNTs

### 2.1. In Vitro Biocompatibility Testing of CNTs

CNTs have been added to cultured cells in in vitro tests and evaluated for their reactions in various cells.

The greatest concern with exposure to nanoparticles in living bodies is its effects on respiratory organs due to inhalation, which was also a problem associated with asbestos exposure. We added multi-walled CNTs (MWCNTs) suspended in 0.1% polyvinyl alcohol (PVA) to V79 Chinese hamster lung fibroblasts that are used in general cytotoxicity tests for the evaluation of cytotoxicity in colony-forming assays. As a control, we used black tattoo ink, composed of carbon nanoparticles, which have been proven to be safe and used throughout human history. Although, the number of colonies of V79 cells decreased according to the concentration of MWCNTs, its cytotoxicity was comparable to tattoo ink. As a result, we found that even if materials are considered safe for living bodies, they may be cytotoxic at high concentrations. Moreover, the MWCNTs used in this study were only as cytotoxic as tattoo ink for V79 cells, exhibiting excellent biocompatibility. Moreover, the evaluation of commercially available tattoo inks showed that most were composed of carbon black, which are carbon nanoparticles for industrial use. Thus, carbon black may be useful as a control for biological safety evaluations of nanoparticles [17].

In addition, we cultured human bronchial epithelial cell lines (BEAS-2B cells) with MWCNTs at concentrations of 1, 10, and 50 μg/mL that were suspended in 0.1% gelatin solution. AlamarBlue assay was used to evaluate cell viability, and the growth of BEAS-2B cells exhibited a concentration-dependent suppression at 24 h after the addition of MWCNTs. Observation by transmission electron microscopy (TEM) showed that MWCNTs were distributed in the lysosomes of the BEAS-2B cell. Flow cytometry was performed using a total reactive oxygen species (ROS)/superoxide detection kit to measure total ROS/superoxide production. As a result, some MWCNTs demonstrated higher oxidative stress and increased activity of superoxide dismutase (SOD) that decomposes active oxygen, compared to the positive control pyocianin, which induces ROS production [18]. Since most materials administered at a high enough dose can cause toxicity, an examination of dosage is necessary. 

Nagai et al. [19] suspended MWCNTs with an approximate diameter of 115 nm at a concentration of 5.0 μg/cm^2^ in 0.5% bovine serum albumin (BSA) solution with human peritoneal mesothelial cells (HPMCs) [20]. Crocidolite, a type of asbestos, was used as a control. After 24 h of culturing, intracellular fibers were observed by confocal microscopy. Although, crocidolite asbestos was observed within cells, MWCNTs were only attached to the cellular surface. The side scatter (SSC) value [21,22] in flow cytometry quantifies the uptake of fibers by cells. SSC values have been reported to increase for crocidolite asbestos, but not for MWCNTs, and the diameter and rigidity of CNTs and crocidolite affect their uptake into cells [19]. Ju et al. [23] conducted an experiment by which MWCNTs were added to human pleural mesothelial cells (MeT-5A). MWCNTs were added in concentrations ranging from 1.25 to 40 μg/cm^2^, and the cell viability showed concentration-dependent suppression at 24 h after treatment. A different study by the same group [24] examined the genotoxicity of MWCNTs by using a γH2AX foci formation technique, wherein MWCNTs were treated within 24 h at a concentration of 10 μg/cm^2^, which did not affect cell viability. Although few γH2AX foci formation was observed by immunofluorescent microscopy within a short 24 h time frame after culturing, the foci formation became more pronounced at 72 h, and the greatest γH2AX foci formation was demonstrated with the longest exposure time at 90 days.

The skin of living organisms in contact with the outside world may have more opportunities to be exposed to nanoparticles. For skin cells, Murray et al. [25] added 1.5 nm diameter, 1–100 μm long single-walled CNTs (SWCNTs) to murine epidermal cells and JB6 P+ cells, and investigated their reaction. SWCNTs with 99.7% purity were added to JB6 P+ cells at 0.06 to 0.24 mg/mL concentration, and cell viability was evaluated by alamarBlue assay at 24 h after treatment. Murray and other research teams have also evaluated NF-κB [26,27,28], a redox-sensitive transcription factor involved in the regulation of many inflammatory responses in the reaction between JB P+ cells and SWCNTs. They also measured the activity of NF-κB with a luminometer which increased with the concentration of SWCNTs, suggesting an association between SWCNTs and inflammatory responses. Furthermore, Patlolla et al. [29] added MWCNTs to normal human dermal fibroblast (NHDF) cells and evaluated cell viability by 3-(4,5-dimethylthiazol-2-yl)-2,5-diphenyl tetrazolium bromide (MTT) assay [30]. When the concentration of MWCNTs was set to 40–400 μg/mL, the cell viability was suppressed, depending on the concentration of MWCNTs. The DNA-damaging effect on NHDF cells was evaluated by comet assay [31]. In this evaluation, there was an increase in the percent tail DNA of NHDF cells, exposed to MWCNTs, demonstrating the genotoxicity of MWCNTs in NHDF cells.

Due to their excellent mechanical strength, CNTs may potentially reinforce the mechanical strength and durability of the original material when used as a composite material. Research has been conducted to improve the performance of composite materials, such as artificial joints and bone fixation materials [6,32]. We evaluated the cells related to the musculoskeletal system, such as bones and joints. In a previous study, we cultured osteoblast-like stromal cells in an osteogenic medium that were collected from the calvaria of 1-day old mice, in order to obtain osteoblasts to be used for experiments. When MWCNTs or carbon black was added to osteoblasts and cultured at a concentration of 0.5 to 50 μg/mL, neither MWCNTs nor carbon black affected the growth of osteoblasts at a concentration of 5 μg/mL. In evaluating calcification using Alizarin Red S staining [33], the group exposed to MWCNTs exhibited better staining compared to carbon black, in addition to significantly higher levels of osteocalcin, a bone formation marker measured by real time PCR [34]. This suggests that MWCNTs, at lower concentrations, and do not exhibit cytotoxicity, may promote bone formation. We also evaluated osteoclasts, which have a role in resorbing the bone matrix. MWCNTs inhibited osteoclast growth, even at a concentration of 5 μg/mL, which did not affect cell growth with osteoblasts. Further verification revealed that MWCNTs inhibited NFATc1 translocation into the nucleus of osteoclast precursor cells in the IκBα/NF-κB pathway when osteoclast precursor cells differentiate into osteoclasts [35]. Based on this result, osteoclasts are less likely to be activated when MWCNTs are used as bone-related biomaterials, and biomaterials may be less likely to loosen or fail. Macrophages in the bone marrow were also evaluated. Macrophage colony-stimulating factor was added to tibial bone marrow cells of ddY mice and cultured to differentiate into macrophages. In addition, MWCNTs were added into the culture, and inflammatory cytokines interleukin (IL)-6, IL-1β, and tumor necrosis factor (TNF)-α in the culture solution were measured by enzyme-linked immunosorbent assay (ELISA). Neither MWCNTs nor tattoo ink increased the inflammatory cytokines in the culture [16]. CNTs have demonstrated favorable osteogenic responses in bone-related cells, and we believe that CNTs can be applied to orthopedic and dental implants and bone regeneration therapy. A summary of the literature discussed in this section is shown in Table 1.

### 2.2. In Vivo Biocompatibility Testing of CNTs

Here, we introduce the in vivo biocompatibility tests in which CNTs are administered to animals in line with clinical medicine.

Mercer et al. [36] administered MWCNTs to C57BL/6J mice by pharyngeal aspiration, and lung tissues were removed 56 days later for histopathological evaluation. Most of the MWCNTs penetrated into alveolar macrophages, but they reported increased thickness due to pulmonary fibrosis and formation of granulomatous lesions. Pulmonary thickness increased over time as the duration of MWCNTs administration increased to 7, 28, and 56 days. Dong et al. [37] also administered MWCNTs to C57BL/6J mice by pharyngeal aspiration. They measured cells and cytokines in broncho-alveolar lavage fluid (BALF). As a result, the BALF of mice exposed to MWCNTs contained a lot of neutrophils, lymphocytes, and macrophages, while ELISA demonstrated many inflammatory cytokines such as IL-6, IL-1β, and TNF-α.

Deng et al. [38] measured the accumulation of intravenously injected ^14^C-labeled MWCNTs in the liver, spleen, and kidney. Many of the intravenously injected MWCNTs were accumulated in the liver and entrapped for 28 days after aspiration. At 90 days, the remaining MWCNTs in the liver were reduced to approximately 20% of the dose. In order to evaluate the effects on the liver, serum lactate dehydrogenase (LDH), total bilirubin (TBIL), total bile acid (TBA), alkaline phosphatase (ALP), and alanine aminotransferase (ALT) were measured. Their bioactivity indices were equivalent to the control and did not affect liver function. Tang et al. [39] also administered MWCNTs intravenously to KunMing mice to assess their effects on the entire body. The blood at one day after MWCNTs administration was evaluated. As a result, administration of MWCNTs did not affect the number of blood cells such as white blood cells (WBC), red blood cells, platelets, bleeding time, and coagulation time show different with the control. Histopathological evaluation of the heart, lung, liver, and kidney, at 1 to 3 days after administration, showed no deposition of MWCNTs and no effect on the tissue. In addition, there were no significant changes to creatine kinase (CK), urea nitrogen, and ALT levels in serologic tests that indicate damage to the myocardium, kidney, and liver, respectively. 

**Figure 2:**
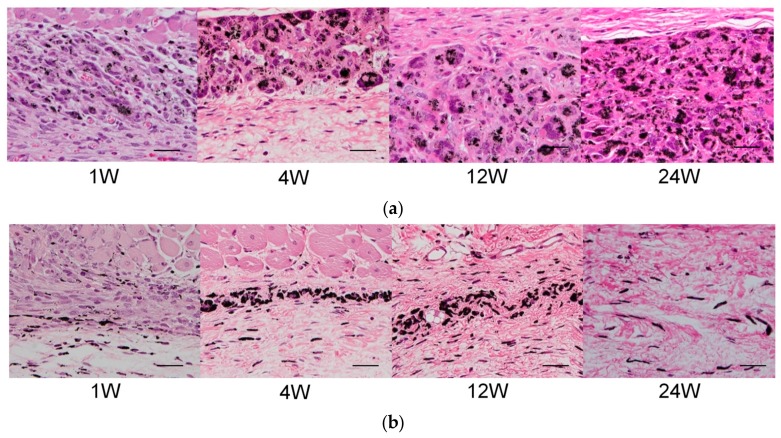
A histopathological image of the skin of ddY mice, subcutaneously administered with MWCNTs. (**a**) Group administered with MWCNT; (**b**) group administered with tattoo ink. In both groups, carbon particles are observed in the macrophages at one week following administration with slight inflammatory cell infiltration around them. At four weeks, carbon particles have remained in the macrophages, and the inflammatory reaction has subsided. Scale bar, 20 μm. Reproduced with permission from [16]. Elsevier, 2011.

We have previously conducted in vivo biocompatibility tests by local administration of MWCNTs. MWCNTs were locally injected into the dorsal subcutaneous tissue of ddY mice, and skin samples were collected and histopathologically evaluated after 1, 4, 12, and 24 weeks of injection with an optical microscope [16]. In both the group administered with MWCNTs and group administered with the tattoo ink control, most of the particles penetrated the macrophages at one week after administration, and there was a mild inflammatory reaction caused by fibroblasts, WBC, and lymphocytes in the surrounding area. At 4 weeks after administration, the inflammatory response subsided and particles remained within the macrophages. Our findings did not change at 12 and 24 weeks after administration (Figure 2), and histological findings in the MWCNTs administration group and the tattoo ink administration group were very similar to common histological findings found in tattooed skin [40].

**Figure 3:**
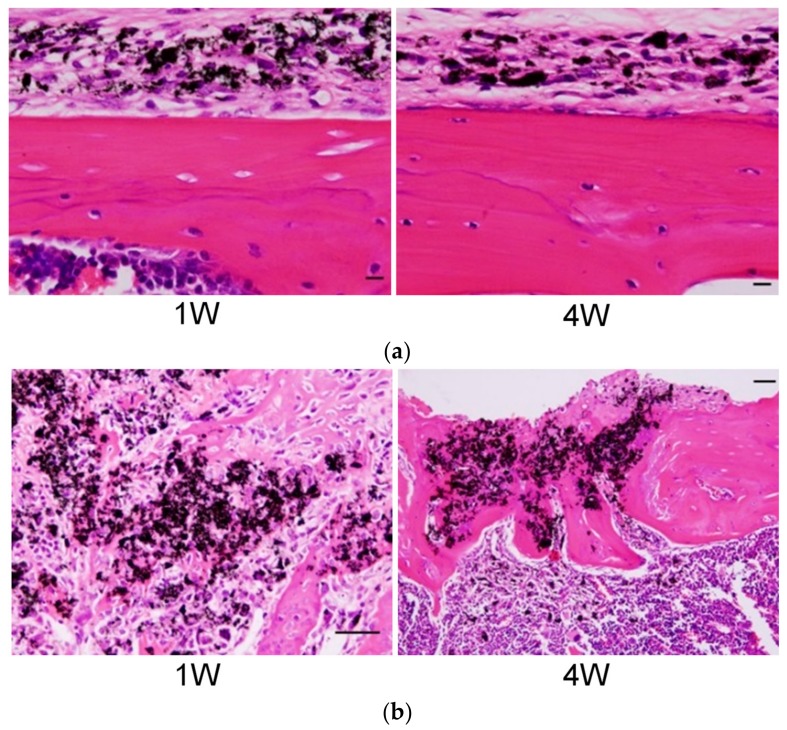
(**a**) Histopathology of ddY mice administered with MWCNTs in the subperiosteum of the skull. MWCNTs were incorporated into macrophages, and no osteolysis was observed in the skull. Scale bar, 20 μm; (**b**) Histopathology of ddY mice, in which MWCNTs were administered into a tibial bone tunnel. One week after administration, woven bone is formed around MWCNTs, and after 4 weeks, bony cortex is regenerated with MWCNTs incorporated into the bone matrix. Scale bar, 20 μm. Reproduced with permission from [41]. John Wiley and Sons, 2008.

In terms of the musculoskeletal system, we have previously administered MWCNTs into bone [41] and within the joint [42]. When MWCNTs were administered to the subperiosteum of the skull in ddY mice, MWCNTs did not cause osteolysis in the skull (Figure 3a). When MWCNTs were administered into a tibial tunnel that was prepared with a drill, the tunnel was filled with new bone formation at 4 weeks after implantation, and MWCNTs were incorporated into the bone matrix (Figure 3b). In addition, there was significantly more bone mineral content in ectopic bone formed under the dorsal fascia of mice with an implant made by combining MWCNTs with recombinant human bone morphogenetic protein-2 (rhBMP-2), compared to an implant that does not contain MWCNTs at 2 weeks after implantation. Similar to the results of in vitro studies, MWCNTs demonstrated a high affinity for bone tissue and promoted bone formation. In addition, after administration of MWCNTs into the knee joint of rats, mild inflammatory cell infiltration was observed in the synovial tissue within one week of administration, and MWCNTs penetrated into macrophages and formed granulation tissues. At 4 and 12 weeks after administration, the inflammatory reaction subsided and granulation tissues remained. These CNTs can be expected to be relatively safe, even when added to biomaterials used in bones and joints.

Although, the type and dosage of CNTs vary from report to report, CNTs generally produce an inflammatory response when administered to the respiratory tract, but have been shown to be relatively safe for intravenous and topical (subcutaneous, intraosseous) administration. A summary of the literature discussed in this chapter is shown in Table 2.

### 2.3. CNT Composites

In recent years, various CNT composites have been created and evaluated to increase the tissue affinity and functional properties of CNTs. Prencipe et al. [43] produced SWCNTs loaded with poly(ethylene glycol) (PEG) chains that were administered intravenously in balb/c mice to measure their blood circulation half-life. PEGylated SWCNTs showed prolonged blood circulation half-life and stability in serum. In addition, Meran et al. [44] evaluated the effects of PEGylated SWCNTs on human umbilical vein endothelial cells (HUVECs). MTT assays were performed using longer SWCNTs with a diameter of 7 Å and length of 120 Å and compared to shorter SWCNTs with a length of 40 Å. Results confirmed that shorter PEGylated SWCNTs did not affect the cell growth of HUVECs, while longer PEGylated SWCNTs suppressed cell growth. Although their mechanisms remain unclear, the addition of substances may introduce new functions and effects to CNTs that are not found in each individual substance.

In addition, a study to improve the function of CNTs by combining poly(ε-caprolactone) (PCL), which is a high polymer evaluated as a bone scaffold. Huang et al. prepared PCL/MWCNT composite scaffolds containing 0.35 wt%, 0.75 wt%, and 3 wt% of MWCNTs, and human adipose-derived stem cells (hADSCs) were cultured on the scaffolds. Scaffolds containing 3 wt% MWCNTs showed the highest cell growth in the alamarBlue assay [45]. Our aforementioned intraosseous implantation test on MWCNTs exhibited high biocompatibility with bone tissue and high osteogenic capacity, and we believe that the MWCNTs promoted bone formation as a PCL scaffold.

Wu et al. [46] created a chitin/MWCNT composite obtained by blending 5 wt% MWCNTs with chitin, a component found in the exoskeleton of crustaceans and insects, and examined the effect on neuron cells (PC12 cells) isolated from the pheochromocytoma of rats. The evaluation of the MTT assay showed that the chitin/MWCNT composite exhibited no cytotoxicity of PC12 cells, and the MWCNT/chitin composite hydrogel was able to retain its morphological integrity longer in the culture solution.

As described above, the biocompatibility of a CNT composite is affected by various factors such as its blended substance, amount of CNTs, and length of CNTs. Even if each blended substance demonstrates excellent biocompatibility, the composite itself should be tested separately as a new material. A summary of the literature discussed in this chapter is shown in Table 3.

### 2.4. Factors Affecting the Biocompatibility Testing of CNTs

Because there are various types of CNTs, we cannot discuss, in general terms, whether CNTs are safe or dangerous for living organisms. The biocompatibility of CNTs is of great interest to many scientists, and various biocompatibility tests have been performed on different types CNTs. This section introduces the factors that affect the biocompatibility of CNTs according to their type.

CNTs are structurally classified into SWCNTs, double-walled CNTs, MWCNTs, and cup-stacked carbon nanotubes (CSCNTs) [47,48]. Nahle et al. [49] evaluated the effect of MWCNTs and SWCNTs on rat alveolar macrophage cell line (NR8383 cells). They added CNTs of various concentrations to NR8383 cells and calculated its 50% inhibitory concentration (IC_50_) by alamarBlue assay. IC_50_ was 4.1 cm^2^/cm^2^ for MWCNTs and 41.2 cm^2^/cm^2^ for SWCNTs, and MWCNTs exhibited a higher cytotoxicity. However, as claimed by these authors, MWCNTs and SWCNTs are different in thickness and length, so the difference in structures may not be the only factor that affects CNTs. We added three different MWCNTs and CSCNTs of different thicknesses and lengths to BEAS-2B cells and human malignant pleural mesothelioma cell line (MESO-1 cells), and evaluated their effects using the alamarBlue assay. In both types of cells, CSCNTs demonstrated higher cell viability than MWCNTs [18].

Zhao et al. [50] evaluated the effects of three different MWCNTs of comparable length (0.5–2 μm) with different diameters on human umbilical vein endothelial cells (HUVECs). The respective diameters of MWCNTs were 10.33 ± 3.50 nm, 21.55 ± 5.66 nm, 38.80 ± and 13.07 nm. IL-6 and ROS were measured by ELISA. IL-6 and ROS showed the highest values in the thinnest MWCNTs, and narrower CNTs showed stronger cytotoxicity. Wang et al. [51] evaluated the effect of CNTs length on biological responses. They compared short and long MWCNTs, with an approximate diameter of 50 nm, and lengths of 0.5–2 μm and 20–50 μm, respectively. When these MWCNTs were added to the mouse leukemic monocyte macrophage cell line (RAW264.7 cells), the production of transforming growth factor (TGF)-β1 that induces extracellular matrix (ECM) protease inhibitors were increased by long MWCNTs. Furthermore, with intra-tracheal administration to spontaneously hypertensive (SH) rats, TGF-β1 in BALF increased in the group administered long MWCNTs, and pulmonary fibrosis was observed in the evaluation of histopathological specimens of the lung.

The purity of CNTs is also known to be associated with biocompatibility, and Murray et al. evaluated cell viability, using SWCNTs containing 0.23% iron impurity, and SWCNTs containing 30% impurity using the aforementioned JB6 P+ cells [25]. In JB6 P+ cells with SWCNTs containing high iron impurity, cell viability was decreased according to alamarBlue assay. The production of antioxidant glutathione also decreased with impure SWCNTs.

Since CNTs present a fibrous morphology, the material tends to tangle. When administered to a living body, it is necessary to disperse these entangled and solidified secondary particles. We dispersed MWCNTs using three types of dispersants to evaluate BEAS-2B cells: Gelatin, carboxylmethyl cellulose (CMC), and 1,2-dipalmitoylsn-glycero-3-phosphocholine (DPPC) [52]. After MWCNT dispersion to the cells, the uptake of MWCNTs into the cells was measured by flow cytometry. At 24 h, the most uptake of MWCNTs into cells was found in the order of gelatin, DPPC, and CMC. In addition, when inflammatory cytokines in the culture medium were measured with a cytometric bead array flex set system, the greatest amount of IL-6 and IL-8 were produced in the order of gelatin, DPPC, and CMC in the same manner as the amount of MWCNTs uptake into the cells. This reveals that the uptake of CNTs into cells is affected by the dispersant, and that cellular response is dependent on the amount of CNT uptake into the cells. A summary of the literature discussed in this chapter is shown in Table 4.

## 3. Carcinogenicity Testing of CNTs

As described above, CNTs are stable fibrous nanoparticles and have been studied for their potential carcinogenicity, given their similarity with asbestos.

Takagi et al. [53] confirmed the formation of mesothelioma in the abdominal cavity following the intraperitoneal administration of MWCNTs from carcinogenesis models consisting of p53^+/−^ mice [54]. No tumor formation occurred in the group intraperitoneally administered with fullerene as a control. In a separate study, they also reported that increasing the dose of MWCNTs increased the rate of mortality for p53^+/−^ mice due to mesothelioma [55].

Suzui et al. [56] evaluated tumor formation in intratracheally administering MWCNTs to F344/Crj rats. None of the animals receiving the control vehicle showed tumor formation, but in the group receiving MWCNTs, 6/38 animals presented with malignant mesothelioma and 14/38 animals exhibited formation of lung tumors (bronchiolo-alveolar adenomas and carcinomas).

**Figure 4:**
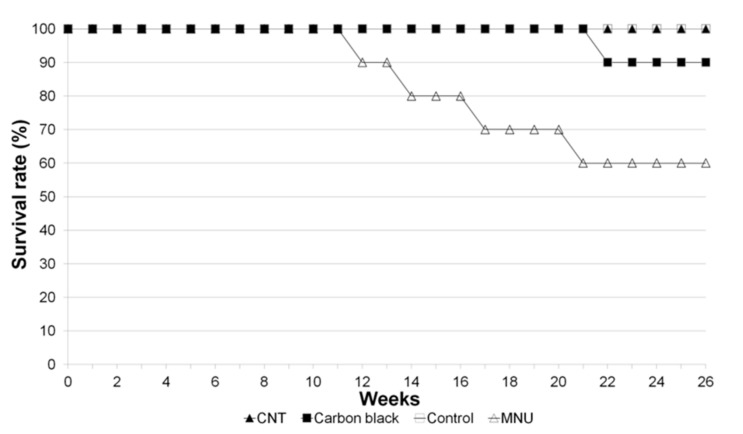
Survival curve of rasH2 mice with local subcutaneous administration of MWCNTs in the dorsum. The survival rate at 26 weeks after administration was 60% in the group that received MNU, a carcinogen, compared to 100% in the group that received MWCNTs. The survival rate of the carbon black group is almost equivalent to tattoo ink at 90%, but the carcinogenic case is likely a coincidence. Image is modified from a study by Takanashi et al. [58].

In order to evaluate carcinogenicity by local administration of CNTs, we subcutaneously administered MWCNTs to rasH2 mice [57], a carcinogenic model mouse. RasH2 mice express human-derived c-H-ras proto-oncogene and die as a result of multi-organ tumors throughout the body after reaching approximately 35 weeks of age. When carcinogenic substances are administered to these mice, tumor formation is accelerated and multi-organ tumors are formed within 26 weeks, which are then used to evaluate the carcinogenicity of the substance. As a control, we used *N*-methyl-*N*-nitrosourea (MNU), a known carcinogen. In the group in which MNU was subcutaneously injected in the dorsum, papilloma and malignant lymphoma of the skin and stomach were observed, and the survival rate at 26 weeks after administration was 60%. However, in the group receiving MWCNTs, there was only one case of inflammatory pseudotumor in the spleen, and the survival rate at 26 weeks after administration was 100% (Figure 4) [58].

**Figure 5:**
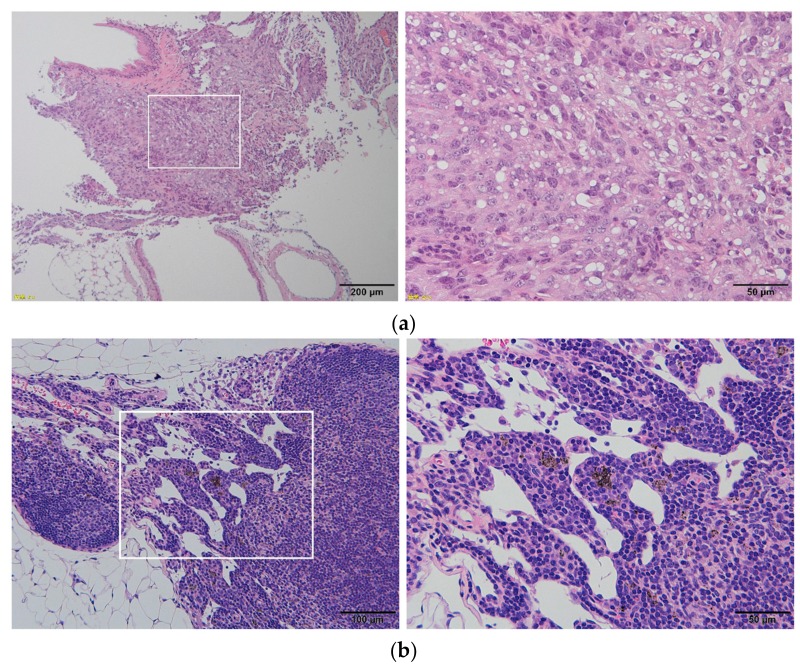
(**a**) Formation of Mesothelioma in the lung of rasH2 mice intravenously administered with MWCNTs. The right is an enlarged image of the square in the left image; (**b**) histological image of the pancreas in rasH2 mice administered intravenously with MWCNTs. Particles of MWCNTs are deposited in the tissue. There is no tumor formation. The right is an enlarged image of the square in the left image. Images are modified from a study by Sobajima et al. [59].

We have also evaluated the effects of systemic intravenously administration of MWCNTs into rasH2 mice [59]. Although, there were models that showed mesothelioma in the lung (Figure 5a) in the group receiving MWCNTs, there was no significant difference in the incidence of tumor with the vehicle group. In searching for MWCNTs using optical microscopy in organs throughout the body, 3.2% of mice showed fiber deposition in pancreas (Figure 5b), but there was no tumor formation in the pancreas.

**Figure 6:**
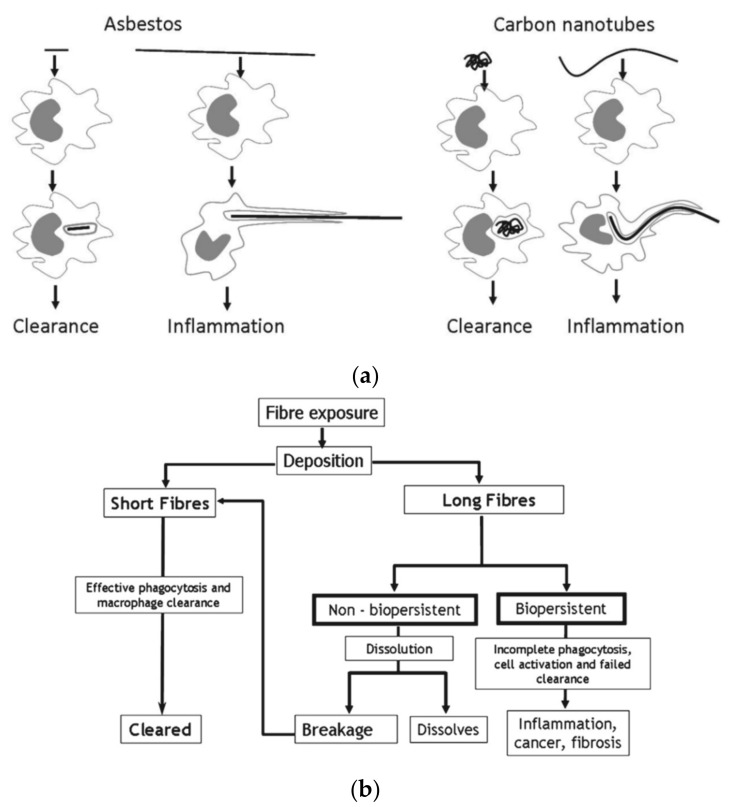
(**a**) When nanofibers such as asbestos (left) and CNTs (right) are phagocytosed by macrophages, inflammation may persist and tumors may be formed if long fibers trigger frustrated phagocytosis. Image is modified from a study by Donaldson et al. [60]. (**b**) Long, biopersistent nanofibers may induce inflammation, cancer, and fibrosis. Reproduced with permission from [61]. Elsevier, 2013.

Donaldson et al. [60] hypothesized a carcinogenic mechanism by which macrophages elicit a persistent inflammatory response when long and thin asbestos, and CNTs are not completely enclosed by macrophage (frustrated phagocytosis), leading to cell tumorigenesis (Figure 6a). In fact, in an experiment in which short and long MWCNTs were administered intraperitoneally to C57BL/6 mice. The formation of granuloma was observed around the MWCNT particles in the peritoneal mesothelium for the long MWCNTs group. Donaldson et al. suggested that stable nanoparticles, with long fibers that are not phagocytosed by macrophages, remain un-degraded in vivo, and that their retention lead to inflammation and subsequent fibrosis and carcinogenesis [61] (Figure 6b). Short fibers and tangled fibers are completely phagocytosed in macrophages, do not cause inflammation, and are unlikely to be tumorigenic. A summary of the literature discussed in this chapter is shown in Table 5.

## 4. Pharmacokinetics of CNTs

When administered into the body, the kinetics of nanoparticles, such as CNTs often remains unclear. Since nanoparticles are small, it is difficult to grasp their distribution and amount in a small quantity of particles. CNTs are also studied for their potential use as carriers in drug delivery systems (DDS) for targeting organs. We believe the pharmacokinetics of CNT administration into the body is an important research topic.

Singh et al. [62] intravenously administered Indium (^111^In)-labeled SWCNTs to BALB/c mice and evaluated the amount of CNTs that translocated to organs and tissues at 30 min, 3 h, and 24 h after administration. At 30 min after administration, CNTs were observed in the blood, femur, liver, kidney, muscle, and skin. However, there was almost no detection after 3 h, and fibers of CNTs were confirmed in excreted urine.

Deng et al. [38] evaluated the pharmacokinetics of ^14^C-tautine-labeled MWCNTs in KunMing mice by intravenous injection, intra-tracheal administration, and oral administration. Many of the MWCNTs were confirmed in the liver after intravenous injection and were entrapped for over 28 days. Intra-tracheally administered MWCNTs were detected in the lung and decreased to approximately 25% after 28 days. Orally administered MWCNTs were gradually excreted in faeces at 12 h after administration via the small and large intestines. In our experiment of intravenous injection of MWCNTs into rasH2 mice, optical microscopy of organs at 26 weeks after administration showed that MWCNTs were deposited only in the pancreas, but no MWCNTs were observed in other organs [59].

In the aforementioned experiment of S p53^+/−^ mice by Takagi et al., MWCNTs were intraperitoneally administered, and singular fibers were observed in the tissues of the choroid plexus at 168 days as well as the lung and kidney at 197 days [53]. Regarding the pharmacokinetics of CNTs, there is no clear agreement between each report, and further research should be conducted.

## 5. Conclusions

Various studies have been conducted on the biocompatibility and carcinogenicity of CNTs. Results have varied widely, as some have reported that the material exhibits high biocompatibility while others have reported that it is carcinogenic. In vivo studies, suggesting the carcinogenicity of CNTs in the literature, were experiments by the inhalation or intraperitoneal administration [53,55,56], and no previous studies have been conducted on intravenous or topical administration. We believe that the carcinogenicity of CNTs is caused by its exposure to mesothelial tissues. Exposure to the abdominal cavity occurs only by artificial administration, but exposure to the respiratory system caused by the dispersion of CNTs into the air. When evaluating CNTs as a material in the industrial field, the aerial dispersion of CNTs during the manufacturing process may risk exposure to the respiratory organs and skin. However, this is an issue that can be overcome by improving the work environment and minimizing the dispersion to undetectable levels. 

When CNTs are used as biomaterials, the effect on the living body varies according to the type of CNTs used, route of administration, and dose. If CNTs are intended to be used in the orthopedic field as a locally implanted biomaterial such as artificial joints or fixation devices, nanoparticle pharmacokinetics remains a problem that warrants further attention. However, there is no need to evaluate the response from their intra-tracheal administration, intraperitoneal administration, and intravenous injection, as safety evaluations for local administration/implantation should be sufficient.

**Figure 7:**
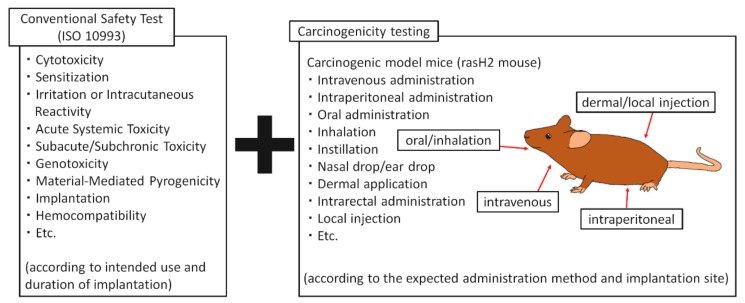
Bio-safety testing of nano-sized biomaterials. Carcinogenicity tests are carried out by using carcinogenic model mice, in addition to conventional safety tests according to the intended application and implantation period of each biomaterial according to ISO 10993-1. The method of administration is selected according to the expected administration method of nano-biomaterials to humans and the implantation site.

When used as reagents for DDS or testing, evaluations should be conducted according to the intended method of administration, such as intravenous administration, intraperitoneal administration, oral administration, inhalation, instillation, nasal drop, dermal application, intra-rectal administration, and local injection. Biological safety assessments of new biomaterials and medical devices are internationally standardized by ISO 10993. Biologic effects, such as cytotoxicity, sensitization, irritation, acute systemic toxicity, and implantation are evaluated according to their intended use and implantation period [63,64]. It is necessary to evaluate the carcinogenicity of nanoparticles, such as CNTs (Figure 7). rasH2 mice can be used to evaluate the carcinogenicity of test materials in a short period of time, and are suitable for testing the carcinogenicity of new biomaterials. By evaluating an animal model, according to the intended use, administration method, and administration site of the new biomaterial, a realistic evaluation can be performed to simulate their clinical use.

CNTs need to be evaluated for their safety according to their type and intended use. In addition to general biological safety tests, it is necessary to perform carcinogenicity tests. If the results of these tests are good, we firmly believe that biological applications of CNTs may be feasible for their use as new high-performance materials. 

## Figures and Tables

**Table 1:** In vitro biocompatibility testing of CNTs.

Author, year	CNT	Cells	Origin of Cells	Control	Evaluations
Hara et al., 2011 [16]	MWCNT	V79 cell	Chinese hamster lung fibroblast	tattoo ink carbon black	colony-foaming assay
Hara et al., 2011 [16]	MWCNT	-	mouse bone macrophage	tattoo ink	IL-1β, IL-6, TNF-α, ELISA
Haniu et al., 2014 [18]	MWCNT	BEAS-2B cell	human bronchial epithelial cell	-	alamarBlue assay total ROS/superpxide detection kit, flow cytometry
Nagai et al., 2011 [35]	MWCNT	-	human peritoneal mesothelial cell	crocidolite	confoal microscopy SSC value, flow cytometry
Ju et al., 2014 [24]	MWCNT	MeT-5A cell	human mesothelial cell	-	γH2AX foci formation technique
Murray et al., 2009 [25]	SWCNT	JB6 P+ cell	murine epidermal cell	-	alamarBlue assay NF-κB actibity, luminometer
Patlolla et al., 2009 [29]	MWCNT	-	normal human dermal fibroblast	-	MTT assay DNA damaging effect, comet assay
Shimizu et al., 2012 [34]	MWCNT	-	mouse calvaria osteoblast-like stromal cell	carbon black	Alizarin Red S stain mRNA of osteocalcin, real time PCR
Narita et al., 2009 [35]	MWCNT	-	mouse osteoclast	carbon black	NFATc1, immunostaining

IL: interleukin, TNF: tumor necrosis factor, ELISA: enzyme-linked immunosorbent assay, ROS: reactive oxygen species, SSC value: side scatter value, MTT assay: (3-(4,5-dimethylthiazol-2-yl)-2,5-diphenyl tetrazolium bromide) assay.

**Table 2:** In vivo biocompatibility testing of CNTs.

Author, year	CNT	Animals	Administration Site/Route	Evaluations
Mercer et al., 2011 [36]	MWCNT	C57BL/6J mouse	pharyngeal aspiration	light microscope examination of lung
Dong et al., 2015 [37]	MWCNT	C57BL/6J mouse	pharyngeal aspiration	IL-6, IL-1β, TNF-α, ELISA of BALF
Deng et al., 2007 [38]	MWCNT	KunMing mouse	intravenously injection	LDH, TBIL, TBA, ALP, ALT in serum (effects to liver)
Tang et al., 2012 [39]	MWCNT	KunMing mouse	intravenously injection	blood count (white blood cells, red blood cells, platelets), bleeding time, coagulation time
Hara et al., 2011 [16]	MWCNT	ddY mouse	subcutaneous tissue	light microscopy
Usui et al., 2008 [41]	MWCNT	ddY mouse	under dorsal fascia	ectopic bone formation
Nomura et al., 2015 [42]	MWCNT	Wistar rat	knee joint	light microscopy

IL: interleukin, TNF: tumor necrosis factor, ELISA: enzyme-linked immunosorbent assay, BALF: bronchoalveolar lavage fluid, LDH: lactate dehydrogenase, TBIL: total bilirubin, TBA: total bile acid, ALP: alkaline phosphatase, ALT: alanine aminotransferase.

**Table 3:** Biocompatibility testing of CNT Composites.

Author, year	CNT	Composite	Cells/Animals	Administration Site/Route	Evaluations
Prencipe et al., 2009 [43]	SWCNT	PEG	balb/c mouse	intravenously injection	circulation time
Meran et al., 2018 [44]	SWCNT	PEG	HUVEC	-	MTT assay
Huang et al., 2019 [45]	MWCNT	PCL	hADSC	-	alamarBlue assay
Wu et al., 2017 [46]	MWCNT	chitin	rat neuron cell	-	MTT assay

PEG: poly (ethylene glycol), HUVEC: human umbilical vein endothelial cell, MTT assay: (3-(4,5-dimethylthiazol-2-yl)-2,5-diphenyl tetrazolium bromide) assay, PCL: poly (ε-caprolactone), hADSC: human adipose derived stem cell.

**Table 4:** Factors affecting the biocompatibility testing of CNTs.

Author, year	CNT	Cells/Animals	Administration Site/Route	Factors	Evaluations
Nahle et al., 2019 [49]	MWCNT SWCNT	NR8383 cell (rat alveolar macrophage cell)	-	MWCNT or SWCNT	alamarBlue assay
Haniu et al., 2014 [18]	MWCNT CSCNT	BEAS-2B cell (human bronchial epithelial cell) MESO-1 cell (human malignant pleural mesothelioma cell)	-	MWCNT or CSCNT	alamarBlue assay
Zhao et al., 2019 [50]	MWCNT	HUVEC	-	diameter of fibers	IL-6, ROS, ELISA
Wang et al., 2013 [51]	MWCNT	RAW264.7 cell (mouse leukemic monocyte macrophage cell) spontaneously hypertensive rat	pharyngeal aspiration	length of fibers	TGF-β1, ELISA light microscope examination of lung TGF-β1, ELISA of BALF
Murray et al., 2009 [25]	SWCNT	JB6 P+ cell (murine epidermal cell)	-	purity of CNT (containing iron)	alamarBlue assay glutathione, fluorescence assay
Haniu et al., 2011 [52]	MWCNT	BEAS-2B cell	-	dispersants (gelatin, DPPC, CMC)	flow cytometry IL-6, IL-8, cytometric bead array flex set system

HUVEC: human umbilical vein endothelial cell, IL: interleukin, ROS: reactive oxygen species, ELISA: enzyme-linked immunosorbent assay, TGF: transforming growth factor, BALF: bronchoalveolar lavage fluid, CMC: carboxylmethyl cellulose, DPPC: 1,2-dipalmitoylsn-glycero-3-phosphocholine.

**Table 5:** Carcinogenicity testing of CNTs.

Author, year	CNT	Animals	Administration Site/Route	Control
Takagi et al., 2008 [53]	MWCNT	p53+/− mouse	intraperitoneal injection	fullerene
Suzui et al., 2016 [56]	MWCNT	F344/Crj rat	pharyngeal aspiration	vehicle
Takanashi et al., 2012 [58]	MWCNT	rasH2 mouse	subcutaneous tissue	MNU
Sobajima et al., 2019 [59]	MWCNT	rasH2 mouse	intravenously injection	MNU

MNU: *N*-methyl-*N*-nitrosourea.

## References

[1] Lu P.J., Cheng W.L., Huang S.C., Chen Y.P., Chou H.K., Cheng H.F. (2015). Characterizing titanium dioxide and zinc oxide nanoparticles in sunscreen spray. Int. J. Cosmet. Sci..

[2] McSweeney P.C. (2016). The safety of nanoparticles in sunscreens: An update for general practice. Aust. Fam. Phys..

[3] Morita T., Takami N. (2006). Nano Si cluster-SiO_x_-C composite material as high-capacity anode material for rechargeable lithium batteries. J. Electrochem. Soc..

[4] Aagaard J. (2004). The Carbomedics aortic heart valve prosthesis: A review. J. Cardiovasc. Surg. (Torino).

[5] Morice M.C., Bestehorn H.P., Carrie D., Macaya C., Aengevaeren W., Wijns W., Dubois C., de Winter R., Verheye S., Hoffmann S. (2006). Direct stenting of de novo coronary stenoses with tacrolimus-eluting versus carbon-coated carbostents. The randomized JUPITER II trial. EuroIntervention.

[6] Saito N., Aoki K., Usui Y., Shimizu M., Hara K., Narita N., Ogihara N., Nakamura K., Ishigaki N., Kato H. (2011). Application of carbon fibers to biomaterials: A new era of nano-level control of carbon fibers after 30-years of development. Chem. Soc. Rev..

[7] Saito N., Haniu H., Usui Y., Aoki K., Hara K., Takanashi S., Shimizu M., Narita N., Okamoto M., Kobayashi S. (2014). Safe clinical use of carbon nanotubes as innovative biomaterials. Chem. Rev..

[8] Jacobsen N.R., Moller P., Clausen P.A., Saber A.T., Micheletti C., Jensen K.A., Wallin H., Vogel U. (2017). Biodistribution of Carbon Nanotubes in Animal Models. Basic Clin. Pharmacol. Toxicol..

[9] Choo H., Jung Y., Jeong Y., Kim H.C., Ku B.C. (2012). Fabrication and Applications of Carbon Nanotube Fibers. Carbon Lett..

[10] Kakooei H., Marioryad H. (2010). Evaluation of exposure to the airborne asbestos in an automobile brake and clutch manufacturing industry in Iran. Regul. Toxicol. Pharmacol..

[11] Luberto F., Ferrante D., Silvestri S., Angelini A., Cuccaro F., Nannavecchia A.M., Oddone E., Vicentini M., Barone-Adesi F., Cena T. (2019). Cumulative asbestos exposure and mortality from asbestos related diseases in a pooled analysis of 21 asbestos cement cohorts in Italy. Environ. Health.

[12] Gogou E., Hatzoglou C., Zarogiannis S.G., Malli F., Jagirdar R.M., Gourgoulianis K.I. (2019). Mesothelioma Mortality Rates in Greece for the Period 2005-2015 Is Increased Compared to Previous Decades. Medicina.

[13] Krowczynska M., Wilk E. (2019). Environmental and Occupational Exposure to Asbestos as a Result of Consumption and Use in Poland. Int. J. Environ. Res. Public Health.

[14] Zha L., Kitamura Y., Kitamura T., Liu R., Shima M., Kurumatani N., Nakaya T., Goji J., Sobue T. (2019). Population-based cohort study on health effects of asbestos exposure in Japan. Cancer Sci..

[15] Carbone M., Yang H. (2017). Mesothelioma: recent highlights. Ann. Transl. Med..

[16] Kane A.B., Hurt R.H., Gao H. (2018). The asbestos-carbon nanotube analogy: An update. Toxicol. Appl. Pharmacol..

[17] Hara K., Aoki K., Usui Y., Shimizu M., Narita N., Ogihara N., Nakamura K., Ishigaki N., Sano K., Haniu H. (2011). Evaluation of CNT toxicity by comparison to tattoo ink. Mater. Today.

[18] Haniu H., Saito N., Matsuda Y., Tsukahara T., Usui Y., Maruyama K., Takanashi S., Aoki K., Kobayashi S., Nomura H. (2014). Biological responses according to the shape and size of carbon nanotubes in BEAS-2B and MESO-1 cells. Int. J. Nanomed..

[19] Nagai H., Okazaki Y., Chew S.H., Misawa N., Yamashita Y., Akatsuka S., Ishihara T., Yamashita K., Yoshikawa Y., Yasui H. (2011). Diameter and rigidity of multiwalled carbon nanotubes are critical factors in mesothelial injury and carcinogenesis. Proc. Natl. Acad. Sci. USA.

[20] Kajiyama H., Shibata K., Terauchi M., Ino K., Nawa A., Kikkawa F. (2008). Involvement of SDF-1alpha/CXCR4 axis in the enhanced peritoneal metastasis of epithelial ovarian carcinoma. Int. J. Cancer.

[21] Stringer B., Imrich A., Kobzik L. (1995). Flow cytometric assay of lung macrophage uptake of environmental particulates. Cytometry.

[22] Al-Jamal K.T., Kostarelos K. (2010). Assessment of cellular uptake and cytotoxicity of carbon nanotubes using flow cytometry. Methods Mol. Biol..

[23] Ju L., Wu W., Yu M., Lou J., Wu H., Yin X., Jia Z., Xiao Y., Zhu L., Yang J. (2017). Different Cellular Response of Human Mesothelial Cell MeT-5A to Short-Term and Long-Term Multiwalled Carbon Nanotubes Exposure. BioMed Res. Int..

[24] Ju L., Zhang G., Zhang X., Jia Z., Gao X., Jiang Y., Yan C., Duerksen-Hughes P.J., Chen F.F., Li H. (2014). Proteomic analysis of cellular response induced by multi-walled carbon nanotubes exposure in A549 cells. PLoS ONE.

[25] Murray A.R., Kisin E., Leonard S.S., Young S.H., Kommineni C., Kagan V.E., Castranova V., Shvedova A.A. (2009). Oxidative stress and inflammatory response in dermal toxicity of single-walled carbon nanotubes. Toxicology.

[26] Driscoll K.E., Carter J.M., Hassenbein D.G., Howard B. (1997). Cytokines and particle-induced inflammatory cell recruitment. Environ. Health Perspect..

[27] Mossman B.T., Churg A. (1998). Mechanisms in the pathogenesis of asbestosis and silicosis. Am. J. Respir. Crit. Care Med..

[28] Schins R.P., Borm P.J. (1999). Mechanisms and mediators in coal dust induced toxicity: A review. Ann. Occup. Hyg..

[29] Patlolla A., Knighten B., Tchounwou P. (2010). Multi-Walled Carbon Nanotubes Induce Cytotoxicity, Genotoxicity and Apoptosis in Normal Human Dermal Fibroblast Cells. Ethn. Dis..

[30] Mosmann T. (1983). Rapid colorimetric assay for cellular growth and survival: application to proliferation and cytotoxicity assays. J. Immunol. Methods.

[31] Olive P.L., Banath J.P., Durand R.E. (1990). Heterogeneity in radiation-induced DNA damage and repair in tumor and normal cells measured using the “comet” assay. Radiat. Res..

[32] Ogihara N., Usui Y., Aoki K., Shimizu M., Narita N., Hara K., Nakamura K., Ishigaki N., Takanashi S., Okamoto M. (2012). Biocompatibility and bone tissue compatibility of alumina ceramics reinforced with carbon nanotubes. Nanomedicine.

[33] Gregory C.A., Gunn W.G., Peister A., Prockop D.J. (2004). An Alizarin red-based assay of mineralization by adherent cells in culture: comparison with cetylpyridinium chloride extraction. Anal. Biochem..

[34] Shimizu M., Kobayashi Y., Mizoguchi T., Nakamura H., Kawahara I., Narita N., Usui Y., Aoki K., Hara K., Haniu H. (2012). Carbon nanotubes induce bone calcification by bidirectional interaction with osteoblasts. Adv Mater..

[35] Narita N., Kobayashi Y., Nakamura H., Maeda K., Ishihara A., Mizoguchi T., Usui Y., Aoki K., Shimizu M., Kato H. (2009). Multiwalled carbon nanotubes specifically inhibit osteoclast differentiation and function. Nano Lett..

[36] Mercer R.R., Hubbs A.F., Scabilloni J.F., Wang L., Battelli L.A., Friend S., Castranova V., Porter D.W. (2011). Pulmonary fibrotic response to aspiration of multi-walled carbon nanotubes. Part Fibre Toxicol..

[37] Dong J., Porter D.W., Batteli L.A., Wolfarth M.G., Richardson D.L., Ma Q. (2015). Pathologic and molecular profiling of rapid-onset fibrosis and inflammation induced by multi-walled carbon nanotubes. Arch. Toxicol..

[38] Deng X., Jia G., Wang H., Sun H., Wang X., Yang S., Wang T., Liu Y. (2007). Translocation and fate of multi-walled carbon nanotubes in vivo. Carbon.

[39] Tang S., Tang Y., Zhong L., Murat K., Asan G., Yu J., Jian R., Wang C., Zhou P. (2012). Short- and long-term toxicities of multi-walled carbon nanotubes in vivo and in vitro. J. Appl. Toxicol..

[40] Ferguson J.E., Andrew S.M., Jones C.J., August P.J. (1997). The Q-switched neodymium:YAG laser and tattoos: a microscopic analysis of laser-tattoo interactions. Br. J. Dermatol..

[41] Usui Y., Aoki N., Narita K., Murakami N., Nakamura I., Nakamura K., Ishigaki N., Yamazaki H., Horiuchi H., Kato H. (2008). Carbon nanotubes with high bone-tissue compatibility and bone-formation acceleration effects. Small.

[42] Nomura H., Takanashi S., Tanaka M., Haniu H., Aoki K., Okamoto M., Kobayashi S., Takizawa T., Usui Y., Oishi A. (2015). Specific biological responses of the synovial membrane to carbon nanotubes. Sci. Rep..

[43] Prencipe G., Tabakman S.M., Welsher K., Liu Z., Goodwin A.P., Zhang L., Henry J., Dai H. (2009). PEG branched polymer for functionalization of nanomaterials with ultralong blood circulation. J. Am. Chem. Soc..

[44] Meran M., Akkus P.D., Kurkcuoglu O., Baysak E., Hizal G., Haciosmanoglu E., Unlu A., Karatepe N., Guner F.S. (2018). Noncovalent Pyrene-Polyethylene Glycol Coatings of Carbon Nanotubes Achieve in Vitro Biocompatibility. Langmuir.

[45] Huang B., Vyas C., Roberts I., Poutrel Q.A., Chiang W.H., Blaker J.J., Huang Z., Bartolo P. (2019). Fabrication and characterisation of 3D printed MWCNT composite porous scaffolds for bone regeneration. Mater. Sci. Eng. C Mater. Biol. Appl..

[46] Wu S., Duan B., Lu A., Wang Y., Ye Q., Zhang L. (2017). Biocompatible chitin/carbon nanotubes composite hydrogels as neuronal growth substrates. Carbohydr. Polym..

[47] Tilmaciu C.M., Morris M.C. (2015). Carbon nanotube biosensors. Front. Chem..

[48] Manawi Y.M., Samara A., Al-Ansari T., Atieh M.A. (2018). A Review of Carbon Nanomaterials’ Synthesis via the Chemical Vapor Deposition (CVD) Method. Materials.

[49] Nahle S., Safar R., Grandemange S., Foliguet B., Lovera-Leroux M., Doumandji Z., Le Faou A., Joubert O., Rihn B., Ferrari L. (2019). Single wall and multiwall carbon nanotubes induce different toxicological responses in rat alveolar macrophages. J. Appl. Toxicol..

[50] Zhao X., Chang S., Long J., Li J., Li X., Cao Y. (2019). The toxicity of multi-walled carbon nanotubes (MWCNTs) to human endothelial cells: The influence of diameters of MWCNTs. Food Chem. Toxicol..

[51] Wang P., Nie X., Wang Y., Li Y., Ge C., Zhang L., Wang L., Bai R., Chen Z., Zhao Y. (2013). Multiwall carbon nanotubes mediate macrophage activation and promote pulmonary fibrosis through TGF-beta/Smad signaling pathway. Small.

[52] Haniu H., Saito N., Matsuda Y., Kim Y.A., Park K.C., Tsukahara T., Usui Y., Aoki K., Shimizu M., Ogihara N. (2011). Effect of dispersants of multi-walled carbon nanotubes on cellular uptake and biological responses. Int. J. Nanomed..

[53] Takagi A., Hirose A., Nishimura T., Fukumori N., Ogata A., Ohashi N., Kitajima S., Kanno J. (2008). Induction of mesothelioma in p53+/− mouse by intraperitoneal application of multi-wall carbon nanotube. J. Toxicol. Sci..

[54] Blanchard K.T., Barthel C., French J.E., Holden H.E., Moretz R., Pack F.D., Tennant R.W., Stoll R.E. (1999). Transponder-induced sarcoma in the heterozygous p53+/− mouse. Toxicol. Pathol..

[55] Takagi A., Hirose A., Futakuchi M., Tsuda H., Kanno J. (2012). Dose-dependent mesothelioma induction by intraperitoneal administration of multi-wall carbon nanotubes in p53 heterozygous mice. Cancer Sci..

[56] Suzui M., Futakuchi M., Fukamachi K., Numano T., Abdelgied M., Takahashi S., Ohnishi M., Omori T., Tsuruoka S., Hirose A. (2016). Multiwalled carbon nanotubes intratracheally instilled into the rat lung induce development of pleural malignant mesothelioma and lung tumors. Cancer Sci..

[57] Mitsumori K., Koizumi H., Nomura T., Yamamoto S. (1998). Pathological features of spontaneous and induced tumors in transgenic mice carrying a human prototype c-Ha-ras gene used for six-month carcinogenicity studies. Toxicol. Pathol..

[58] Takanashi S., Hara K., Aoki K., Usui Y., Shimizu M., Haniu H., Ogihara N., Ishigaki N., Nakamura K., Okamoto M. (2012). Carcinogenicity evaluation for the application of carbon nanotubes as biomaterials in rasH2 mice. Sci. Rep..

[59] Sobajima A., Haniu H., Nomura H., Tanaka M., Takizawa T., Kamanaka T., Aoki K., Okamoto M., Yoshida K., Sasaki J. (2019). Organ accumulation and carcinogenicity of highly dispersed multi-walled carbon nanotubes administered intravenously in transgenic rasH2 mice. Int. J. Nanomed..

[60] Donaldson K., Murphy F.A., Duffin R., Poland C.A. (2010). Asbestos, carbon nanotubes and the pleural mesothelium: A review of the hypothesis regarding the role of long fibre retention in the parietal pleura, inflammation and mesothelioma. Part Fibre Toxicol..

[61] Donaldson K., Poland C.A., Murphy F.A., MacFarlane M., Chernova T., Schinwald A. (2013). Pulmonary toxicity of carbon nanotubes and asbestos—Similarities and differences. Adv. Drug Deliv. Rev..

[62] Singh R., Pantarotto D., Lacerda L., Pastorin G., Klumpp C., Prato M., Bianco A., Kostarelos K. (2006). Tissue biodistribution and blood clearance rates of intravenously administered carbon nanotube radiotracers. Proc. Natl. Acad. Sci. USA.

[63] ISO 10993-1:2018. Biological Evaluation of Medical Devices—Part 1: Evaluation and Testing within a Risk Management Process. https://www.iso.org/standard/68936.html.

[64] ISO 10993-3:2014. Biological Evaluation of Medical Devices—Part 3: Tests for Genotoxicity, Carcinogenicity and Reproductive Toxicity. https://www.iso.org/standard/55614.html.

